# Saliva profiling with differential scanning calorimetry: A feasibility study with ex vivo samples

**DOI:** 10.1371/journal.pone.0269600

**Published:** 2022-06-10

**Authors:** Lena Pultrone, Raphael Schmid, Tuomas Waltimo, Olivier Braissant, Monika Astasov-Frauenhoffer

**Affiliations:** 1 Clinic for Oral Health & Medicine, University Center for Dental Medicine Basel UZB, University of Basel, Basel, Switzerland; 2 Center of Biomechanics and Biocalorimetry, c/o Department of Biomedical Engineering (DBE), University of Basel, Allschwil, Switzerland; 3 Department Research, University Center for Dental Medicine Basel UZB, University of Basel, Basel, Switzerland; Universidad de Granada, SPAIN

## Abstract

Differential scanning calorimetry (DSC) has been used widely to study various biomarkers from blood, less is known about the protein profiles from saliva. The aim of the study was to investigate the use DSC in order to detect saliva thermal profiles and determine the most appropriate sampling procedure to collect and process saliva. Saliva was collected from 25 healthy young individuals and processed using different protocols based on centrifugation and filtering. The most effective protocol was centrifugation at 5000g for 10 min at 4°C followed by filtration through Millex 0.45 μm filter. Prepared samples were transferred to 3 mL calorimetric ampoules and then loaded into TAM48 calibrated to 30°C until analysis. DSC scans were recorded from 30°C to 90°C at a scan rate of 1°C/h with a pre-conditioning the samples to starting temperature for 1 h. The results show that the peak distribution of protein melting points was clearly bimodal, and the majority of peaks appeared between 40–50°C. Another set of peaks is visible between 65°C– 75°C. Additionally, the peak amplitude and area under the peak are less affected by the concentration of protein in the sample than by the individual differences between people. In conclusion, the study shows that with right preparation of the samples, there is a possibility to have thermograms of salivary proteins that show peaks in similar temperature regions between different healthy volunteers.

## Introduction

Over the years, body fluids have been providing an excellent base for creating diagnostic tools as they contain various different proteins and other biomolecules. As blood is circulating through all organs—including those with disease—and its collection is well-standardized, that makes blood components by far the most common choice for diagnostics [1].

Saliva has foremostly, important biological functions such as lubrification and the cleansing of the oral cavity, the facilitating of the speech, assistance of the taste, mastication and swallowing, start of digestion [2]; it also contains a mixture of proteins such as mucins, amylases, defensins, cystatins, histatins, proline-rich proteins, statherin, lactoperoxidase, lysozyme, lactoferrin, and immunoglobulins [3] that are secreted from multiple salivary glands (parotid, submandibular, sublingual and other minor glands) [4].

The term “salivaomics” was introduced in 2008 to highlight the rapid development of knowledge about various “omics” constituents of saliva, including: proteome, transcriptome, micro-RNA, metabolome, and microbiome. Since then, new technologies and a wide range of salivary biomarkers have been validated to make the use of saliva a clinical reality [5]. More than 100 salivary biomarkers (DNA, RNA, mRNA, proteins) in oral cancer detection have already been identified, e.g cytokines [6]. However, previous studies have confirmed that also many discriminatory salivary biomarkers can be detected upon the development of systemic cancers such as pancreatic [7], breast [8], and lung cancer [9].

Spielmann and Wong compared the protein compositions from human salivary and plasma fluids and found that even though these fluids have less intersection of the same proteins, the molecular mechanism, biological processes, and cellular elements show similarity [10]. However, in comparison to blood, saliva has important advantages as a diagnostic fluid: it can be collected without any help of health professionals [2, 4], in a stress-free non-invasive way without difficulties and many opportunities [11]. This can be crucial for people with mental disorders, children or elderly, where obtaining blood samples can be difficult [12]. Furthermore, storage and transportation have lower costs [5, 11] and sufficient quantities for analysis are given, as healthy individuals have a daily salivary secretion of up to 1.5L [13].

Due to the abundance of studies focusing on salivary biomarkers, it is not easy to discover novel disease markers; however, it is useful to apply different methods that allow possible detection of changes in the proteome even before clinical signs appear [14, 15]. Garbett et al. [16] were able to reveal changes in the thermal profiles of major plasma proteins with differential scanning calorimetry (DSC) analysis from healthy individuals and patients with different diseases. Indeed, DSC has gone a long way since its development around fifty years ago. In early studies, mainly large proteins in high concentration were analysed and the focus was primarily on the process of protein folding rather than make use of it in modern medicine [17]. However, information is gained about the thermal stability of the biomolecules as DSC is able to measure and reveal all small changes in the heat capacity of protein while undergoing temperature changes [18–20]. Now being an elaborate method in research, many different human proteins are being examined, such as monoclonal antibodies or fibrinogen [21–23]. Moreover, many studies have concentrated the effort to investigate changes in protein thermograms to be able to diagnose chronic pulmonary disease [24], type 1 diabetis [25], glioblastoma [26, 27], melanoma with regional lymph node or distal metastases [28], breast cancer [29], colorectal cancer [30], and cervical cancer [31]. However, until now, no studies have focused on evaluating thermograms of protein profiles of saliva by DSC.

These comparisons between saliva and serum and the fact, that saliva is readily available, should be enough to investigate whether saliva can be used to produce new relevant protein markers using DSC. Thus, the aim of the study was to determine the most appropriate sampling procedure to collect and prepare saliva and investigate the use DSC in order to detect saliva thermal profiles of healthy volunteers to evaluate the feasibility of the method.

## Results

**Fig 1:**
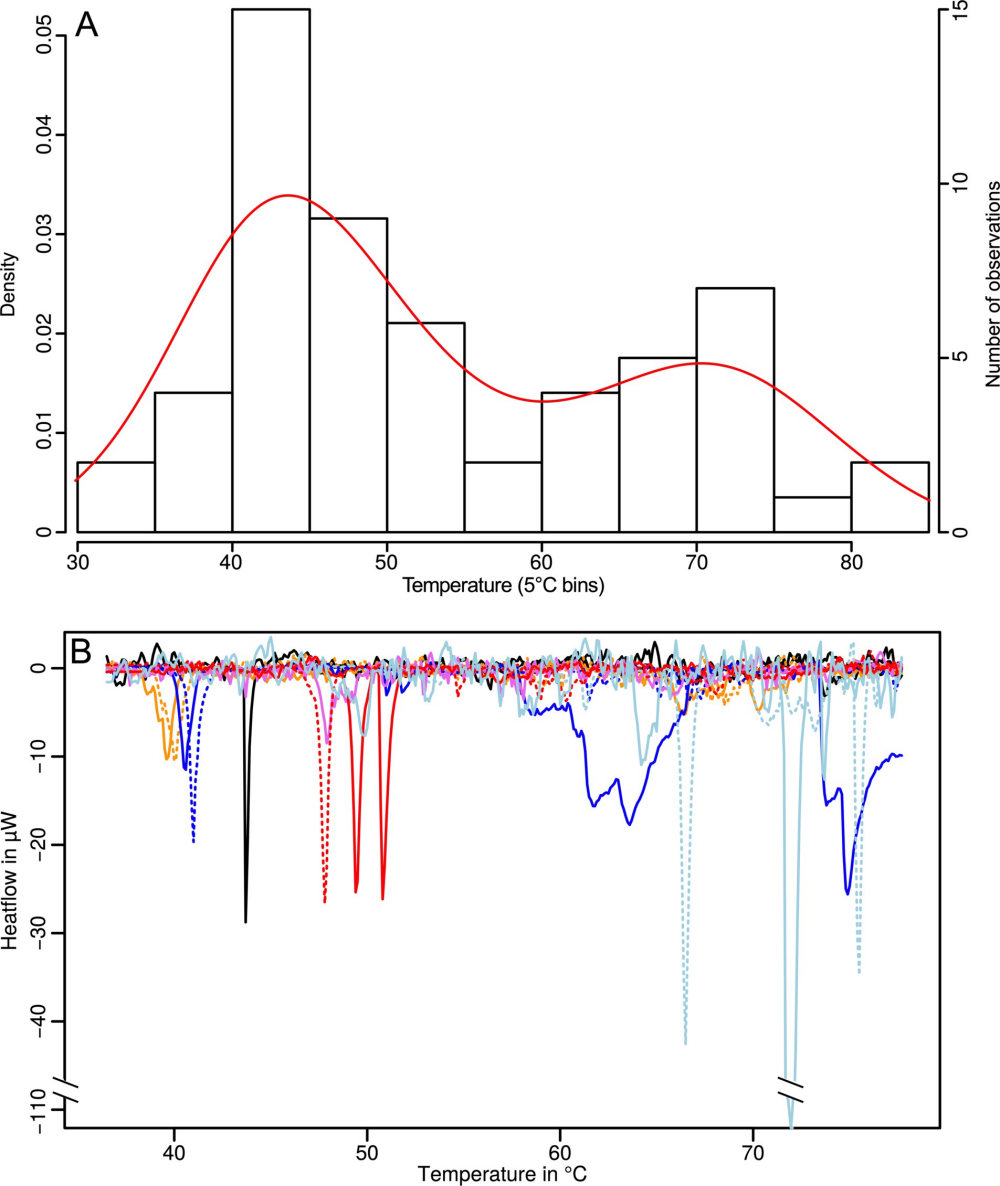
Distribution of proteins by denaturation temperature. (A) Peak distribution in saliva samples in 5°C intervals for all healthy volunteers tested; (B) DSC pattern from the same healthy volunteer taken different days (colour refers to the sample collected at the same time; dashed and solid lines show the replicates for a sample collected and treated the same way).

Although different saliva preparation protocols were used, thermograms were obtained only with protocol IV, where centrifugation for 10 minutes at 5000 g and filtering through a Millex filter with a pore size of 0.45 μm were applied. The other protocols resulted in the presence of bacteria in the saliva sample (average CFU/mL for protocols I-III was 2x10^3^, 3.5x10^4^, and 2.4x10^3^, respectively), or an absence of DSC thermograms due to the loss or degradation of the proteins (V-VI). Both stimulated and unstimulated saliva was collected as it is to be expected that unstimulated saliva has a slightly higher concentration of proteins and thus, might lead to better sample analysis. However, no statistically significant differences were seen in the protein concentrations between these two groups (average concentration stimulated vs average concentration unstimulated; 0.91 ± 0.41 mg/mL vs 0.94 ± 0.37 mg/mL, respectively). Thus, only stimulated saliva profiles are presented in Table 1 as this is the more convenient way from the perspective of sample collection for the donors. Parameters for thermal profiles of saliva with protocol IV are shown in Table 1. The peak distribution was clearly bimodal (Fig 1A) and the majority of peaks appeared between 40°C-50°C. Another set of peaks is visible between 65°C-75°C. No correlations were found between the concentration of proteins and peak temperature values (r = 0.13, p = 0.34). Additionally, the peak amplitude and area (under the peak) are less affected by the concentration of protein (r = -0.23, p = 0.09 and r = 0.17, p = 0.19, respectively) in the sample than by the individual differences between people. Indeed, there was a rather high variability even in a single volunteer due to the daily variations in the saliva composition and amount (Fig 1B).

**Table 1:** Parameters of thermal profiles obtained from stimulated saliva samples.

Temperature range	Peak temperature (°C)	Peak heatflow (μW)	Enthalpy (mJ)	Concentration (mg/mL)
**30°C–40°C**	32.1	-8.4	-15.12	0.87 ± 0.27
	34	-10.53	-17.85	0.95 ± 0.01
	39.4	-28.4	-32.55	1.25 ± 0.01
	39.4	-23.41	-23.76	0.54 ± 0.01
	39.5	-14.28	-21.01	0.54 ± 0.08
	39.8	-4.8	-24.3	0.35 ± 0.02
**40°C–50°C**	40.3	-11.7	-37	0.40 ± 0.06
	40.5	-6.5	-8.6	0.45 ± 0.11
	40.9	-11.6	-23.75	0.46 ± 0.01
	41	-18.37	-20.09	0.47 ± 0.01
	41.1	-17.36	-29.09	0.74 ± 0.01
	41.2	-16.1	-25.8	1.17 ± 0.16
	41.2	-7.33	-15.2	0.77 ± 0.02
	41.3	-8.6	-14.6	1.02 ± 0.02
	41.4	-10.74	-26.86	0.88 ± 0.01
	41.6	-5.34	-15.2	0.77 ± 0.02
	42	-10.8	-11.2	0.91 ± 0.05
	43.6	-8.13	-12.5	0.65 ± 0.02
	43.6	-8.68	-11.6	0.95 ± 0.09
	43.9	-37.4	-22.9	0.33 ± 0.05
	44	-25.4	-39.4	1.37 ± 0.07
	45.1	-9.14	-7.15	1.48 ± 0.03
	45.1	-4.8	-6.5	0.74 ± 0.07
	45.8	-28.3	-54.8	0.44 ± 0.04
	46.7	-9.6	-17.3	2.10 ± 0.01
	46.9	-13.3	-22	0.83 ± 0.03
	47.5	-23.9	-53.4	1.32 ± 0.30
	48.1	-27.3	-35.9	0.64 ± 0.05
	49	-29.8	-39.5	0.83 ± 0.01
	49.5	-24.1	-32	0.81 ± 0.03
**50°C–60°C**	50.6	-19.1	-22.9	0.84 ± 0.07
	51	-26.4	-40.6	0.81 ± 0.03
	51.6	-30.6	-31.7	1.07 ± 0.50
	52.1	-38	-50.7	1.37 ± 0.50
	53	-52.5	-69.9	0.66 ± 0.01
	53.8	-44.1	-41	1.35 ± 0.03
	58	-22.7	-24.2	1.22 ± 0.24
	58.8	-25.7	-19.8	1.17 ± 0.16
**60°C–70°C**	60.9	-46.8	-134.7	0.69 ± 0.04
	62.4	-32.6	-46.2	1.14 ± 0.19
	63.8	-90.5	-72.5	1.45 ± 0.29
	64.2	-12.9	-16.5	0.50 ± 0.01
	66	-38.45	-26.35	0.68 ± 0.01
	68.2	-66.7	-68.6	2.10 ± 0.01
	68.3	-22.5	-26	0.56 ± 0.01
	69.3	-33.9	-39.6	0.65 ± 0.09
	69.8	-21.7	-41.1	0.82 ± 0.10
**70°C–80°C**	70.7	-21.7	-20.7	0.45 ± 0.02
	71	-136.06	-91.94	0.71 ± 0.04
	73.5	-20.7	-19.6	0.98 ± 0.01
	73.5	-66.8	-40.2	1.14 ± 0.19
	74.2	-42.7	-19.3	0.78 ± 0.03
	74.5	-42.7	-17.4	0.78 ± 0.03
	74.6	-57.2	-49.2	1.13 ± 0.04
	76	-93.87	-34.31	1.04 ± 0.18
**80°C–90°C**	80.2	-24.1	-26.6	0.47 ± 0.02
	81.2	-12.2	-11.4	0.49 ± 0.02

**Fig 2:**
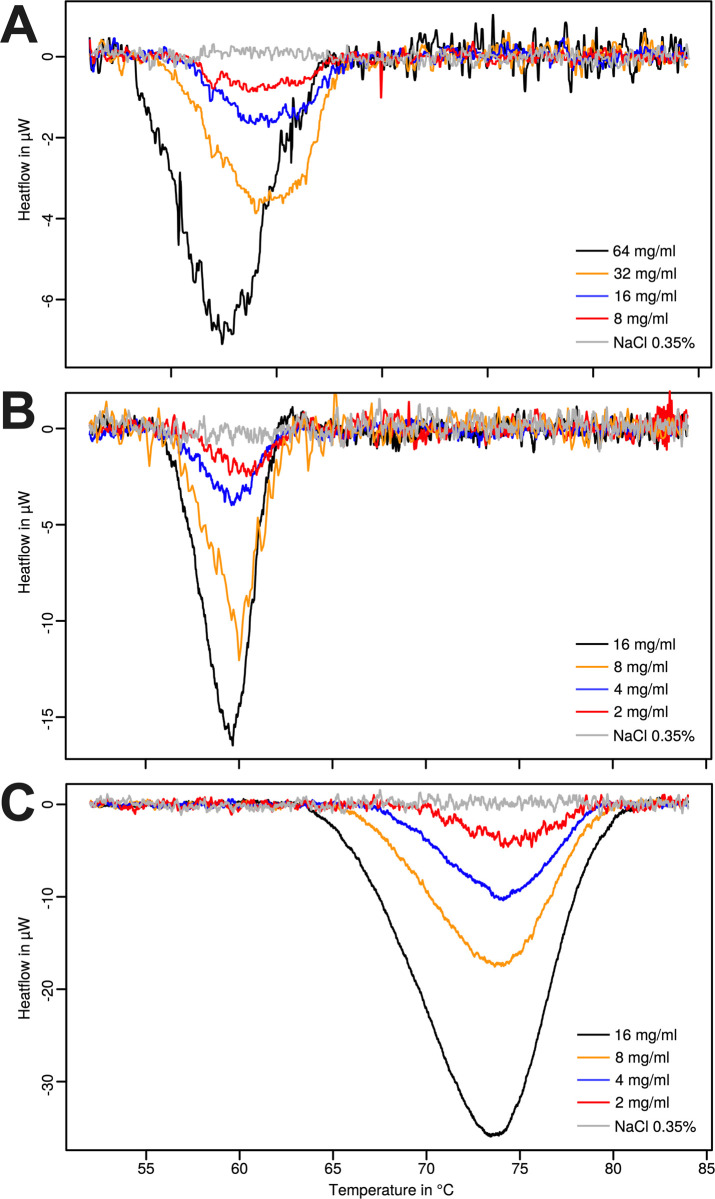
DSC pattern of increasing concentrations of protein measured in the TAM 48: (A) Mucin, (B) bovine serum albumin, (C) lysozyme. Peak value and enthalpy measured can be found in Table 2.

Results for standard proteins obtained are shown in Fig 2 and Table 2. These results are in line with their known melting temperatures found in the literature. For these protein standards, the concentration of the protein did show a strong correlation to the parameters of the thermal profiles. For lysozyme and BSA very good correlations were obtained. The maximum heatflow correlated well with the concentration (r = -0.99 and r = -0.98 for lysozyme and BSA respectively–see data in Table 2). Similarly, the enthalpy measured also correlated well (r = -0.99 and r = -0.99 for lysozyme and BSA respectively–see data in Table 2). For mucin, signal was much lower and there was a bit more spread in the data measured the correlation between the peak heatflow measured and concentration was r = -0.94. Also, here a correlation between the peak heatflow measured and concentration of mucin of r = -0.97 was observed. Overall, the measurement with standard protein confirms the accuracy and the possible use of TAM48 DSC for such application. All correlation were significant (p<0.05).

**Table 2:** Data showing the main DSC peak parameters (enthalpy, peak heatflow and peak temperature) for the standard protein tested.

	Concentration [mg/ml]	Enthalpy [mJ]	Peak heatflow [μW]	Peak temperature[°C]	n
**BSA**	16	-231 ± 20	-18.4 ± 0.7	59.7 ± 0.1	5
**BSA**	8	-118 ± 14	-11.8 ± 1.2	59.7 ± 0.2	5
**BSA**	4	-54 ± 14	-5.2 ± 1.0	60.1 ± 0.4	5
**BSA**	2	-20 ± 7	-2.2 ± 0.5	59.9 ± 0.6	3
**BSA [32]**				58.8–59.8	
**BSA [33]**				59.8–60.9	
**Lysozyme**	16	-932 ± 7	-33.7 ± 1.5	73.5 ± 0.4	5
**Lysozyme**	8	-460 ± 8	-17.9 ± 1.7	74.0 ± 0.4	5
**Lysozyme**	4	-209 ± 5	-9.1 ± 1.8	74.2 ± 0.3	5
**Lysozyme**	2	-104 ± 2	-5.9 ± 1.1	74.7 ± 0.6	5
**Lysozyme [34]**		-1373 ± 28	NA	73.8 ± 0.1	
**Lysozyme [35]**		-1072 ± 6	NA	76.7 ± 0.1	
**Mucin**	64	-37 ± 5	-7.2 ± 1.4	58.7 ± 0.7	6
**Mucin**	32	-19 ± 4	-4.3 ± 1.2	59.2 ± 0.8	6
**Mucin**	16	-11 ± 2	-2.5 ± 0.5	59.9 ± 0.8	6
**Mucin**	8	-5 ± 1	-1.4 ± 0.4	59.7 ± 0.8	3
**Saline**	NA	0 ± 1	-0.9 ± 0.9^§^	NA	6

^**§**^ Indicate the short-term noise rather than a specific peak

## Discussion

Blood plasma has been used to detect various diseases based on the overall thermograms determined by DSC [22, 36]. However, saliva provides a protein profile like this found in blood plasma and, therefore could be a valuable addition to biomarker collection to differentiate between healthy and disease. Additionally, collecting saliva samples is of course easier than having samples of blood from persons; however, that only applies for healthy people with normal salivary flow. People suffering from dry mouth or other similar conditions might not be able to provide enough volume to be analysed [2]. Another factor that makes analysing saliva more complicated than blood products is that saliva contains also of high number of bacteria and ca 30 times less protein [37]. Moreover, sample preparation includes purification step so that only the fraction containing salivary, and no bacterial proteins is assessed. Thus, different protocols were used in this study to evaluate their efficacy on the removal of bacteria. While only centrifugation was not enough to eliminate the bacteria, excessive filtering on the other hand led to loss of salivary proteins and no distinctive peaks to be detected in DSC thermograms. In the end the fraction collected by centrifugation followed by one filtering step was the only one that allowed to obtain thermal profiles from experiment to experiment most likely due to sufficient amount of proteins.

In order to optimise the signal and receive more reliable results from DSC, a higher concentration of proteins in the solution would be desirable. Indeed, compared to plasma where the average protein concentration ranges between 60–80 mg/mL [38] the range reported for salivary proteins only reaches values comprised between 0.67 to 2.37 mg/mL [39] and only up to 2.1 mg/mL in the present study. It is known that saliva contains only about 0.3% proteins while over 99% of the solution is water [10]. Therefore, two different protocols (V-VI) were used to increase the protein concentration in the samples of this study. Unfortunately, the protocols used here to increase the concentration while decreasing the volume, were not able to keep the temperature stable enough to avoid protein denaturation. Thus, a more intensive analysis on how to maintain the integrity of the proteins needs to be assessed maybe by using buffering system or other protocols for increasing the concentration.

The results reveal that most of the peaks we found were between 30°C– 50°C, which corresponds to the knowledge that salivary proteome contains a larger proportion (14.5%) of low molecular weight proteins, mainly <20kDa. In comparison only 7% of plasma proteome is in that size range. In total, up to 65% of salivary proteins have a molecular weight under 65kDa, while in serum that proportion is only 36%. Additionally, there is a fraction of proteins (27%) with is found common between saliva and plasma, and their molecular weight distributions are similar to the distributions of the salivary proteome with a tendency toward the low-molecular-weight end, except in the ≥200 kDa range [40].

Also, the correlation between the protein concentration and parameters assessed by DSC was in the scope of this study. However, no strong correlation was detected in any of the temperature range groups for the salivary samples. That could be caused by the presence of other molecules that either interact with protein (stabilizing them) or denaturating or reacting at same temperature; thus, perturbing the signal. The concentration of the control proteins did show a strong correlation to the parameters of the thermal profiles; thus, the weak correlation of saliva samples was not due to the handling or detection, but due to the physico-chemical properties of the sample. During this project many physico-chemical parameters such as handling time, age of the chemicals, as well as mathematical parameters such as baseline correction were shown as possible factors that could affect the quality of the data. This was exemplified by the lower reproducible of the measurement of early measurements of standard proteins leading to reasonable values but with much higher spread (see S1 Table). Thus, this should encourage researchers using DSC to use fresh chemicals and to reduced handling time as much as possible.

It is important to also note that although the calorimeter used in this study allows to process several samples at the same time and was very helpful to establish this proof-of-concept study, other calorimeters such as nano-DSC or Flash DSC could provide better alternatives using smaller volumes of sample. Additionally, due to their rapid temperature change these instruments can process sample rather fast and still maintaining a good throughput. Moreover, the saliva samples could benefit from fast scanning rate as it reduces chances of unspecific protein degradation (by proteases that might be present in the sample).

In conclusion, although saliva is easy to collect, the proteins are very sensitive to temperature changes before the measurement and thus, an optimal buffering system might be able to help with this problem and needs to be assessed in more detail. However, the study shows the first time that thermograms of salivary proteins are showing peaks in similar temperature regions between different healthy volunteers and DSC could be considered as a method for further detailed examinations on salivary proteome. Additionally, proteomic data might help to further assign the peaks observed to proteins or peptides that could eventually later on be used as biomarkers.

## Materials & methods

### Preparation of samples

Altogether 25 healthy young volunteers participated in this study (24.9y ± 3.9y). All of them were verbally informed about the study and upon agreeing to participate, their verbal consent was registered together with their age. All volunteers were instructed to not eat or drink anything at least 2h prior to donation of unstimulated as well as stimulated saliva (by chewing paraffin tablets) like it is common practise at a dental check-up. All leftover samples were discarded by the end of the study. All sample collection performed for this study involving human volunteers was in accordance with the ethical standards of the institutional and national research committee and with the 1964 Helsinki declaration and its later amendments or comparable ethical standards.

All samples were stored constantly on ice. The saliva samples were then prepared using different protocols adapted of knowledge gained through thorough literature search to eliminate all possible bacterial counterpart from the samples: (I) centrifugation at 6000 g for 20 min at 4°C followed by centrifugation at 16200 g for 30 min at 4°C; (II) centrifugation at 20000 g for 30 min at 4°C; (III) centrifugation at 5000 g for 10 min at 4°C followed by filtration through Millex 5 μm filter; (IV) centrifugation at 5000 g for 10 min at 4°C followed by filtration through Millex 0.45 μm filter; (V) same as (IV) followed by concentration through Amicon® Ultra 0.3 mL Ultracel® membrane; (VI) same as (IV) followed by lyophilization (Integrated SpeedVac System ISS110, Savant, Fischer Scientific AG, Reinach, Switzerland). All protocols were screened twice and as only protocol IV revealed reliable results (the other five protocols did not allow any peaks to be detected), it was repeated for three more times (n = 5).

The concentration of proteins in processed saliva was assessed by Bradford protein assay and absorption measured at OD_595_ (Synergy HT Multi-Detection Microplate Reader, BioTek^®^, Luzern, Switzerland). Due to the viscosity of saliva samples, they were split into three aliquots to assure concentration measurements were correct (n = 3).

Standard proteins (bovine serum albumin, mucin from bovine submaxillary glands, lysozyme, paraffin; all from Sigma Aldrich, Buchs, Switzerland) were weighed to match various concentrations in sterile saline solution as shown in Table 2.

### Differential scanning calorimetry

Saliva samples were transferred to 4 mL calorimetric ampoules and then loaded into TAM48 calibrated to 30°C until analysis. DSC scans were recorded from 30°C to 90°C at a scan rate of 1°C/h with a pre-conditioning the samples to starting temperature for 1 h. Duplicate DSC scans were obtained for each sample to assure no drop-outs due to single sample failure (for example: non-optimal closing of an ampoule). Different saliva preparation protocols were repeated twice altogether thereafter, most optimized protocol was repeated for five times. Standard protein samples were analysed using the same procedure but with a temperature range between 50 and 85°C. All samples were analysed for three parameters: peak temperature (°C), peak amplitude (μW), and peak integral (mJ). Data analysis was performed using the manufacturer software (TAM assistant), Fityk (https://fityk.nieto.pl/), and with R version 3.4.4. Normality of all the parameters was checked by the Shapiro-Wilk test for small sample size. Parametric (Pearson correlation) was used for normally distributed data from samples with reference compounds (Table 2). Data of healthy volunteers were not normally distributed and thus, non-parametric test (Spearman correlation) was used to estimate the correlation between concentration of the samples and the different thermogram parameters. All the statistical analysis were performed using GraphPad Prism version 9.3.1 for MacOS, GraphPad Software, San Diego, California USA, www.graphpad.com.

## Supporting information

S1 Table High variation found in measured parameters of thermal profiles obtained for various standard proteins to verify the suitability of the method that was due to many physico-chemical parameters such as handling time, age of the chemicals, as well as mathematical parameters such as baseline correction were shown as possible factors that could affect the quality of the data. (DOCX)
