# Supplementary material for: Saliva profiling with differential scanning calorimetry: A feasibility study with ex vivo samples
Source: PLoS One. 2022 Jun 10;17(6):e0269600. doi: 10.1371/journal.pone.0269600 (PMC9187081; doi:10.1371/journal.pone.0269600)
Supplement: S1 Table — (DOCX) [file pone.0269600.s001.docx]

| Protein | Peak temperature (ºC) | Peak amplitude (µW) | Peak integral (mJ) | Concentration (mg/mL) |  |
| --- | --- | --- | --- | --- | --- |
|  |  |  |  |  |  |
| BSA | 67.20 ± 0.01 | -7.20 ± 1.46 | - 59.33 ± 8.95 | 8 |  |
|  | 65.03 ± 0.38 | -4.27 ± 1.70 | -23.46 ± 14.34 | 4 |  |
|  | 65.8 | -1.26 | -14.26 | 2 |  |
|  | 66.3 ± 1.08 | -22.33 ± 6.14 | 11.51 ± 3.98 | 0.125 |  |
| Lysozyme | 78.50 ± 0.01 | -11.83 ± 1.32 | -207.57 ± 24.09 | 8 |  |
|  | 77.15 ± 0.92 | -2.78 ± 0.78 | -36.23 ± 26.96 | 4 |  |
| Mucin | 45.00 ± 1.27 | -6.01 ± 4.48 | -122.37 ± 3.28 | 8 |  |
|  | 71.1 | -1.26 | -30 | 4 |  |
|  | 76 | -93.87 | -34.31 | 1 |  |
|  | 66.7 | -22.7 | -28 | 0.5 |  |
| Parafin | 56.11 ± 0.01 | -98.45 ± 13.08 | -1500.41 ± 706.93 | 29.3 |  |
